# Predictors of Hospitalized Exacerbations and Mortality in Chronic Obstructive Pulmonary Disease

**DOI:** 10.1371/journal.pone.0158727

**Published:** 2016-06-30

**Authors:** Miguel Santibáñez, Roberto Garrastazu, Mario Ruiz-Nuñez, Jose Manuel Helguera, Sandra Arenal, Cristina Bonnardeux, Carlos León, Juan Luis García-Rivero

**Affiliations:** 1 Preventive Medicine and Public Health Area, Universidad de Cantabria-IDIVAL, Santander, Cantabria, Spain; 2 Centro de Salud de Gama, Servicio Cántabro de Salud, Bárcena de Cicero, Cantabria, Spain; 3 Centro de Salud de Liérganes, Servicio Cántabro de Salud, Miera, Cantabria, Spain; 4 Centro de Salud Bajo Asón, Servicio Cántabro de Salud, Cantabria, Ampuero, Spain; 5 Centro de Salud de Suances, Servicio Cántabro de Salud, Suances, Cantabria, Spain; 6 Centro de Salud Campoo-Los Valles, Servicio Cántabro de Salud, Mataporquera, Cantabria, Spain; 7 Centro de Salud de Suances, Servicio Cántabro de Salud, Suances, Cantabria, Spain; 8 Pneumology Department, Hospital de Laredo, Servicio Cántabro de Salud, Laredo, Cantabria, Spain; National Institute for Public Health and the Environment, NETHERLANDS

## Abstract

**Background and Aim:**

Exacerbations of chronic obstructive pulmonary disease (COPD) carry significant consequences for patients and are responsible for considerable health-care costs—particularly if hospitalization is required. Despite the importance of hospitalized exacerbations, relatively little is known about their determinants. This study aimed to analyze predictors of hospitalized exacerbations and mortality in COPD patients.

**Methods:**

This was a retrospective population-based cohort study. We selected 900 patients with confirmed COPD aged ≥35 years by simple random sampling among all COPD patients in Cantabria (northern Spain) on December 31, 2011. We defined moderate exacerbations as events that led a care provider to prescribe antibiotics or corticosteroids and severe exacerbations as exacerbations requiring hospital admission. We observed exacerbation frequency over the previous year (2011) and following year (2012). We categorized patients according to COPD severity based on forced expiratory volume in 1 second (Global Initiative for Chronic Obstructive Lung Disease [GOLD] grades 1–4). We estimated the odds ratios (ORs) by logistic regression, adjusting for age, sex, smoking status, COPD severity, and frequent exacerbator phenotype the previous year.

**Results:**

Of the patients, 16.4% had ≥1 severe exacerbations, varying from 9.3% in mild GOLD grade 1 to 44% in very severe COPD patients. A history of at least two prior severe exacerbations was positively associated with new severe exacerbations (adjusted OR, 6.73; 95% confidence interval [CI], 3.53–12.83) and mortality (adjusted OR, 7.63; 95%CI, 3.41–17.05). Older age and several comorbidities, such as heart failure and diabetes, were similarly associated.

**Conclusions:**

Hospitalized exacerbations occurred with all grades of airflow limitation. A history of severe exacerbations was associated with new hospitalized exacerbations and mortality.

## Introduction

Chronic obstructive pulmonary disease (COPD) is one of the most prevalent lung diseases observed in clinical practice and the third-leading cause of death in the world [1]. The association of COPD with smoking is well established. However, an increasing number of studies have reported a considerable prevalence of COPD among nonsmokers [2,3,4].

Exacerbations of COPD are characterized by episodes of symptom worsening. If case definition is made according to health-care utilization criteria, exacerbations are defined as events that lead a care provider to prescribe antibiotics, corticosteroids, or both (moderate exacerbations) or that result in hospitalization (severe exacerbations) [5,6]. Exacerbations of COPD carry significant consequences for patients [7,8]; they are responsible for a large proportion of the health-care costs attributable to this prevalent condition [9]—particularly if they require hospitalization [10]. Consequently, preventing exacerbations is a key component of COPD-management strategies [11,12].

Despite the importance of exacerbations, relatively little is known about their determinants; this is probably because the heterogeneity of COPD exacerbations reflects their dependence on a complex spectrum of multiple risk factors [6,13]. Recently, new large observational cohort studies, such as the Evaluation of COPD Longitudinally to Identify Predictive Surrogate Endpoints (ECLIPSE) study [14], have shown that the most consistent predictor of exacerbations appears to be a previous history of exacerbations; this potentially indicates a definable phenotype of exacerbation susceptibility.

Epidemiology and determinants specifically associated with exacerbations that require hospital admission have been less extensively described [15,16,17,18]. The ECLIPSE study has shown that exacerbations requiring hospital admission occur across all grades of airflow limitation and are a significant prognostic factor of overall mortality. The main predictor of exacerbations requiring hospital admission was a past history of similar events (exacerbations requiring hospital admission the previous year). The severity of the underlying COPD has also been shown to be an independent predictor of higher risk of exacerbations requiring hospital admission and reduced survival [18]. However, the ECLIPSE study did not include milder forms of COPD (mild Global Initiative for Chronic Obstructive Lung Disease [GOLD] grade 1). It also did not include confirmed COPD patients with no history of tobacco consumption.

We used data from a retrospective population-based cohort study, including mild GOLD grade 1 and non-smoking patients with confirmed COPD, to test predictors of severe exacerbations and overall mortality in COPD.

## Materials and Methods

### Ethics Statement

We obtained approval of the research protocol from the Clinical Research Ethics Committee of Cantabria before data acquisition. Prior to analysis, patient records and information were anonymized and de-identified.

### Design and Participants

This was a retrospective population-based cohort study. All the patients were ≥35 years old, with prevalent codes of R91 or R95 according to the International Classification of Primary Care [19]. We identified them from electronic clinical databases in the province of Cantabria (northern Spain) on December 31, 2011. The recruitment criteria included no restrictions regarding history of tobacco consumption or degree of forced expiratory volume in 1 second (FEV1) impairment. Of the total population of 362,372 people registered in Cantabria, we identified 9,334 potential COPD patients.

We obtained a sample of 2000 patients by simple random sampling. We carefully examined the spirometric data for each of these patients. COPD diagnosis was considered as confirmed if a spirometry with bronchodilator test consistent with obstructive disease (defined as FEV1/forced vital capacity ratio of <0.7) was retrieved from the databases; we rejected the diagnosis if the spirometry test result was not consistent with obstructive disease. We confirmed the diagnosis of COPD in 900 patients (45.3%) and rejected it in 197 patients (9.9%); with the remainder (44.8%), it was neither confirmed nor rejected. We restricted our final analysis to confirmed COPD patients.

### Data Sources and Variables

We obtained the data for each patient from computerized clinical databases of primary health-care centers and hospital records. The clinical and demographic characteristics of all the patients were recorded, including age, years since diagnosis of COPD, treatments and vaccinations, smoking status, alcohol consumption, and comorbidities.

We used the same the case definition of exacerbation as that in the ECLIPSE cohort study [14] according to health-care utilization criteria [5,6]. Thus, moderate exacerbations were defined as events that led a care provider to prescribe antibiotics, corticosteroids, or both; severe exacerbations were defined as those requiring hospital admission. We observed the exacerbation frequency over the previous year (2011) and following year (2012). We defined frequent exacerbations (frequent exacerbator phenotype) as two or more exacerbations in a year, including both moderate and severe exacerbations; we did so because this definition coincides with current health-care utilization criteria for frequent exacerbations [6,14].We also categorized patients according to the severity of their level of airflow limitation (GOLD grades 1–4) [11].

### Statistical Analyses

We expressed categorical and discrete variables as counts (percentage) and continuous variables as mean and standard deviation [SD]. We assessed statistical differences between groups using the chi-square or Fisher’s exact test, where appropriate, for categorical variables. We used the Student’s *t* test for the continuous variables.

We treated severe exacerbation frequency for the following year as a dichotomous dependent variable in the regression models: no severe exacerbations versus one or more severe exacerbations during 2012. We computed overall mortality for all causes during 2012, and we treated it as a dichotomous dependent variable in the regression models, classifying patients into survivors and non-survivors. We treated overall exacerbation frequency (moderate and severe exacerbations) during the previous year (2011) as an ordinal variable (0 exacerbations, 1 exacerbation, ≥2 exacerbations). We also ordinally categorized exacerbation frequency restricted to severe exacerbations (0 exacerbations, 1 severe exacerbation, and ≥2 severe exacerbations). We ordinally categorized patients into four categories according to the severity of COPD (FEV1 GOLD grades 1–4). Lastly, FEV1 was transformed on an ordinal scale according to 5% incremental decreases in the percentage of the predicted value [14].

As an association measure, we used the odds ratio. Odds is a frequency measure; the odds of some event reflect the likelihood that the event will occur. The relative odds or odds ratio (OR) in a cohort study is simply the odds of the event in the exposed group divided by the odds of event in the unexposed group. To prevent misunderstanding with respect to the OR, we use the term “odds” instead of “risk” because the OR may sometimes tend to overestimate the risk ratio or relative risk [20,21].

We estimated crude and adjusted ORs with their 95% confidence intervals (95%CI) by unconditional logistic regression. We adjusted for the following: age (continuous); sex; smoking status (non-smoker, former smoker, current smoker); severity of COPD (ordinal GOLD grades 1–4); and frequent exacerbator phenotype (yes/no) the previous year.

We calculated tests for OR trends for the ordinal independent variables using logistic models that included categorical terms as continuous variables. For these trend tests, we used the likelihood ratio test.

The alpha error was set at 0.05, and all *p* values were bilateral. We conducted all statistical analyses using IBM SPSS Statistics version 22.0.

## Results

The baseline characteristics of the patients appear in Table 1. Among the 900 patients with confirmed COPD, 194 (21.6%) were women. The overall mean age was 71.2 years [SD 11.0], with a mean of 6.6 years [SD 5.5] since diagnosis of COPD. Of the confirmed COPD patients, 15.7% had never smoked.

**Table 1:** Baseline sociodemographic, lifestyle, and clinical characteristics of the patients.

	Hospitalized Exacerbations								
	None^a^			≥1			Total		
	N = 752	row %	Column%	N = 148	row %	Column%	N = 900	Column%	*p value*
**Age**, Mean [SD]	70.4	[11.1]		75.1	[9.59]		71.2	[11.0]	*<0*.*001*
***Gender***									
Female	174	89.7%	23.1%	20	10.3%	13.5%	194	21.6%	*0*.*009*
Male	578	81.9%	76.9%	128	18.1%	86.5%	706	78.4%	
***Year since CODP diagnosis*,** Mean [SD]	6.4	[5.4]		7.8	[5.9]		6.6	[5.5]	*0*.*005*
***Smoking***									
Never smoker	119	88.1%	16.6%	16	11.9%	10.9%	135	15.7%	*<0*.*001*
Former smokers	390	78.2%	54.5%	109	21.8%	74.1%	499	57.9%	
Current smokers	206	90.4%	28.8%	22	9.6%	15.0%	228	26.5%	
*Missing*	37	97.4%	4.9%	1	2.6%	0.7%	38	4.2%	
***Body mass index (BMI)***									
Normal weight (18.5–24.9)	129	84.9%	19.0%	23	15.1%	17.8%	152	18.8%	*0*.*668*
Overweight (25–29.9)	260	83.9%	38.3%	50	16.1%	38.8%	310	38.4%	
Obesity (≥30)	283	84.2%	41.7%	53	15.8%	41.1%	336	41.6%	
Underweight (<18.5)	7	70.0%	1.0%	3	30.0%	2.3%	10	1.2%	
*Missing*	73	79.3%	9.7%	19	20.7%	12.8%	92	10.2%	
**GOLD Grades (FEV1)**									
Grade 1 (≥80%)	68	90.7%	11.5%	7	9.3%	5.3%	75	10.4%	*<0*.*001*
Grade 2 (≥50–80%)	363	83.8%	61.4%	70	16.2%	53.0%	433	59.9%	
Grade 3 (≥30–49,9%)	146	76.8%	24.7%	44	23.2%	33.3%	190	26.3%	
Grade 4 (<30%)	14	56.0%	2.4%	11	44.0%	8.3%	25	3.5%	
*Missing*	161	91.0%	21.4%	16	9.0%	10.8%	177	19.7%	
**History of exacerbations the previous year**									
None (0 exacerbations)	303	93.2%	40.3%	22	6.8%	14.9%	325	36.1%	*<0*.*001*
1 exacerbation	203	85.3%	27.0%	35	14.7%	23.6%	238	26.4%	
≥2 exacerbations	246	73.0%	32.7%	91	27.0%	61.5%	337	37.4%	
**History of severe exacerbations (requiring Hospital admission) the previous year**									
None (0 COPD Admissions)	649	88.9%	86.3%	81	11.1%	54.7%	730	81.1%	*<0*.*001*
1 COPD Admission	79	68.7%	10.5%	36	31.3%	24.3%	115	12.8%	
≥2 COPD Admissions	24	43.6%	3.2%	31	56.4%	20.9%	55	6.1%	* *

“Column%” denotes the valid percentage (without counting missing values).

In decreasing order of COPD severity, the patients were as follows: moderate GOLD grade 2, 60%; severe GOLD grade 3, 26.2%; mild grade 1, 10.4%; and very severe GOLD grade 4, 3.5%. Of the mild GOLD grade 1 patients, 9.3% developed at least one hospitalized exacerbation the following year. The incidence of at least one hospitalized exacerbation increased in our study with increasing disease severity: 16.2%, 23.2%, and 44.0% in GOLD grades 2, 3, and 4, respectively.

In of our sample, 85% of patients were affected by at least one of the studied comorbidities. The most prevalent comorbidity was high blood pressure, which affected 60.2% of the confirmed COPD patients (S1 Table).

Table 2 shows the association between a history of exacerbations and the risk of hospitalized exacerbations and mortality the following year. Independent of COPD severity, a history of hospitalized exacerbations the previous year was positively associated with new severe exacerbations: adjusted OR ≥2 severe exacerbations, 6.73; 95%CI, 3.53–18.83 (adjusted linear *p* trend <0.001). Overall exacerbations (moderate and severe) were also associated with new severe exacerbations: adjusted OR ≥2 exacerbations 3.74; 95%CI, 2.17–6.43 (adjusted linear *p* trend <0.001).

**Table 2:** Crude and adjusted odds ratios (ORs) according to previous exacerbations for the risk of hospitalized exacerbations and mortality the following year.

	Hospitalized Exacerbations								Mortality^d^							
	None ^a^	≥1							Survivors	Non-Survivors						
	N = 752	N = 148	ORc^b^	(95%	CI)	ORa^c^	(95%	CI)	N = 831	N = 69	ORc^b^	(95%	CI)	ORa^c^	(95%	CI)
**Number of exacerbations the previous year**																
None (0 exacerbations)	303	22	1	—		1	—		313	12	1	—		1	—	
1 exacerbation	203	35	2.38	1.35	4.17	1.53	0.81	2.87	214	24	2.93	1.43	5.98	3.5	1.39	8.77
≥2 exacerbations	246	91	5.1	3.11	8.36	3.74	2.17	6.43	304	33	2.83	1.44	5.59	2.95	1.22	7.09
*Linear p trend*			*<0*.*001*			*<0*.*001*					0.001			0.03		
**Number of severe exacerbations**																
None (0 COPD Admissions)	649	81	1	—		1	—		695	35	1	—		1	—	
1 COPD Admission	79	36	3.65	2.31	5.77	3.12	1.88	5.18	97	18	3.69	2.01	6.76	4.15	2.00	8.6
≥2 COPD Admissions	24	31	10.35	5.79	18.5	6.73	3.53	12.83	39	16	8.15	4.15	15.98	7.63	3.41	17.05
*Linear p trend*			*<0*.*001*			*<0*.*001*					*<0*.*001*			*<0*.*001*		

^a^ “None” denotes 0 COPD admissions the following year. “≥1” denotes at least one COPD admission the following year.

^b^ Odds ratios and 95% confidence intervals: “ORc” denotes crude odds ratio.

^c^ “ORa” denotes adjusted OR by age, sex, smoking status, and COPD severity (GOLD Grades 1–4).

^d^ Overall mortality for all causes.

Independent of COPD severity, a history of at least two hospitalized exacerbations the previous year was also associated with overall mortality: adjusted OR, 7.63; 95%CI, 3.41–17.05.

Table 3 shows the association according to COPD severity for the risk of hospitalized exacerbations and mortality the following year.

**Table 3:** Crude and adjusted odds ratios (ORs) according to COPD severity for the risk of hospitalized exacerbations and mortality the following year.

	Hospitalized Exacerbations										
	None ^a^	≥1									
	**N = 752**	**N = 148**	**ORc^b^**	**(95%**	**CI)**	**ORa1^c^**	**(95%**	**CI)**	**ORa2^d^**	**(95%**	**CI)**
**COPD severity according to FEV1**											
FEV1—per 5% decrease in % of predicted value	—	—	1.16	1.09	1.23	1.15	1.07	1.22	1.12	1.05	1.20
FEV1—Mild-GOLD Grade 1 (reference category)	68	7	1	—		1	—		1	—	
FEV1—Moderate-GOLD Grade 2	363	70	1.87	0.83	4.25	1.55	0.67	3.59	1.31	0.56	3.08
FEV1—Severe-GOLD Grade 3	146	44	2.93	1.25	6.83	2.35	0.98	5.60	1.85	0.76	4.50
FEV1- Very Severe-GOLD Grade 4	14	11	7.63	2.52	23.13	6.57	2.10	20.52	4.39	1.36	14.11
*Linear p trend*			*0*.*01*			*<0*.*001*			0.005		
*Missing*	*161*	*16*	*0*.*97*	*0*.*38*	*3*.*45*	*0*.*99*	*0*.*37*	*2*.*61*	*0*.*99*	*0*.*37*	*2*.*65*
	**Mortality**^e^		** **	** **	** **	** **	** **	** **	** **	** **	** **
	**Survivors**	**Non-survivors**	** **	** **	** **	** **	** **	** **	** **	** **	** **
	**N = 831**	**N = 69**	**ORc**^**b**^	**(95%**	**CI)**	**ORa1**^**c**^	**(95%**	**CI)**	**ORa2**^**d**^	**(95%**	**CI)**
**COPD severity according to FEV1**											
FEV1—per 5% decrease in % of predicted value	—	—	1.04	0.98	1.12	1.01	0.94	1.10	1.00	0.91	1.10
FEV1—Mild-GOLD Grade 1 (reference category)	69	6	1	—		1	—		1	—	
FEV1—Moderate-GOLD Grade 2	399	34	0.98	0.4	2.42	0.91	0.33	2.50	0.87	0.31	2.40
FEV1—Severe-GOLD Grade 3	177	13	0.84	0.31	2.31	0.75	0.25	2.29	0.70	0.23	2.16
FEV1- Very Severe-GOLD Grade 4	21	4	2.19	0.56	8.5	2.41	0.56	10.48	2.16	0.49	9.44
*Linear p trend*			*0*.*683*			*0*.*704*			*0*.*807*		
*Missing*	*165*	*12*	*0*.*84*	*0*.*3*	*2*.*32*	*1*.*05*	*0*.*34*	*3*.*24*	*1*.*09*	*0*.*35*	*3*.*39*

^a^ “None” denotes 0 COPD admissions the following year. “≥1” denotes at least one COPD admission the following year.

^b^ Odds ratios and 95% confidence intervals: “ORc” denotes crude odds ratio

^c^ “ORa1” denotes adjusted OR by age, sex, and smoking status.

^d^ “ORa2” denotes adjusted OR added to the multivariable model: frequent exacerbator phenotype (yes/no) the previous year.

^e^ Overall mortality for all causes.

More severe airflow limitation was associated with a higher odds of new hospitalized exacerbations the following year, and it showed a statistically significant dose-response trend: adjusted OR GOLD grade 4, 6.57; 95%CI, 2.10–20.52; linear *p* trend <0.001. After adding the frequent exacerbator phenotype (yes/no) the previous year to the multivariable model, the associations diminished; however, the statistical significance remained: adjusted OR GOLD grade 4, 4.39; 95%CI, 1.36–14.11.

COPD severity was associated with a higher odds of mortality, but without yielding statistical significance: adjusted OR GOLD grade 4, 2.16; 95%CI, 0.49–9.44.

With respect to the sociodemographic (age, sex) and lifestyle (tobacco, body mass index) variables, only age was statistically significantly associated with a higher odd of hospitalized exacerbations and mortality the following year in the multivariable models (Table 4). Regarding comorbidities, heart failure, atrial fibrillation, any severe heart disease, diabetes, and lung cancer were statistically significantly associated with both hospitalized exacerbations and mortality the following year (Table 5).

**Table 4:** Crude and adjusted odds ratios (ORs) according to sociodemographic and lifestyle characteristics for the risk of hospitalized exacerbations and mortality the following year.

	Hospitalized Exacerbations								Mortality^d^							
	None ^a^	≥1							Survivors	Non-Survivors						
	N = 752	N = 148	ORc^b^	(95%	CI)	ORa^c^	(95%	CI)	N = 831	N = 69	ORc^b^	(95%	CI)	ORa^c^	(95%	CI)
**Age**-per 10-years increase	—	—	1.44	1.21	1.71	1.35	1.09	1.68	—	—	1.87	1.44	2.44	1.08	1.04	1.12
***Gender***																
Female	174	20	1	—		1	—		183	11	1	—		1	—	
Male	578	128	1.93	1.17	3.18	1.77	0.94	3.33	648	58	1.49	0.77	2.90	1.94	0.69	5.45
***Smoking***																
Never smoker	119	16	1	—		1	—		128	7	1	—		1	—	
Former smokers	390	109	2.08	1.18	3.65	1.55	0.77	3.11	459	40	1.59	0.70	3.64	1.84	0.58	5.85
Current smokers	206	22	0.79	0.40	1.57	0.94	0.41	2.13	211	17	1.47	0.60	3.65	3.15	0.90	10.95
*Linear p trend*			*0*.*163*			*0*.*539*					*0*.*53*			*0*.*045*	
***Body mass index (BMI)***																
Normal weight (18.5–24.9)	129	23	1	—		1	—		142	10	1	—		1	—	
Overweight (25–29.9)	260	50	1.08	0.63	1.85	0.92	0.49	1.72	283	27	1.36	0.64	2.88	1.20	0.50	2.84
Obesity (≥30)	283	53	1.05	0.62	1.79	0.98	0.53	1.83	316	20	0.90	0.41	1.97	0.58	0.23	1.50
Underweight (<18.5)	7	3	2.40	0.58	9.98	2.03	0.37	11	9	1	1.58	0.18	13.73	2.23	0.20	24.61
*Linear p trend*			*0*.*668*			*0*.*766*					*0*.*639*			*0*.*223*		

^a^ “None” denotes 0 COPD admissions the following year. “≥1” denotes at least one COPD admission the following year.

^b^ Odds ratios and 95% confidence intervals: “ORc” denotes crude odds ratio

^c^ “ORa” denotes adjusted OR by age, sex, smoking status, COPD severity (GOLD Grades 1–4) and frequent exacerbator phenotype (yes/no) the previous year.

^d^ Overall mortality for all causes

**Table 5:** Crude and adjusted odds ratios (ORs) according to comorbidities for the risk of hospitalized exacerbations and mortality the following year.

	Hospitalized Exacerbations								Mortality^f^							
	None^a^	≥1							Survivors	Non-Survivors						
	N = 752	N = 148	ORc^b^	(95%	CI)	ORa^c^	(95%	CI)	N = 831	N = 69	ORc^b^	(95%	CI)	ORa^c^	(95%	CI)
**Co-morbidities**																
None	126	9	1	—		1	—		131	4	1	—		1	—	
At least one of the co-morbidities below	626	139	3.11	1.54	6.27	3.32	1.41	7.84	700	65	3.04	1.09	8.49	1.66	0.55	4.98
**Atrial Fibrillation**																
No	626	99	1	—		1	—		682	43	1	—		1	—	
Yes	126	49	2.46	1.66	3.64	1.78	1.12	2.85	149	26	2.77	1.65	4.65	2.01	1.06	3.82
**Ischemic heart disease**																
No	632	111	1	—		1	—		698	45	1	—		1	—	
Yes	120	37	1.76	1.15	2.67	1.29	0.79	2.09	133	24	2.80	1.65	4.75	1.67	0.86	3.24
**Heart Failure**																
No	632	90	1	—		1	—		687	35	1	—		1	—	
Yes	120	58	3.39	2.31	4.98	3.13	2.00	4.91	144	34	4.63	2.80	7.68	3.74	2.01	6.98
**Any severe heart disease** ^d^																
No	498	58	1	—		1	—		537	19	1	—		1	—	
Yes	254	90	3.04	2.12	4.37	2.56	1.67	3.94	294	50	4.81	2.78	8.31	3.41	1.77	6.57
**High Blood Pressure**																
No	303	55	1	—		1	—		335	23	1	—		1	—	
Yes	449	93	1.14	0.79	1.64	0.88	0.57	1.36	496	46	1.35	0.80	2.27	0.95	0.51	1.78
**Diabetes**																
No	559	93	1			1			612	40	1	—		1	—	
Yes	193	55	1.71	1.18	2.48	1.79	1.17	2.74	219	29	2.03	1.23	3.35	1.98	1.09	3.59
**Osteoporosis**																
No	685	131	1	—		1	—		755	61	1	—		1	—	
Yes	67	17	1.33	0.75	2.33	1.55	0.80	3.00	76	8	1.30	0.60	2.82	1.23	0.44	3.44
**Psychiatric History**																
No	503	90	1	—		1	—		545	48	1	—		1	—	
Yes	249	58	1.30	0.91	1.87	1.42	0.92	2.19	286	21	0.83	0.49	1.42	0.73	0.37	1.42
**Lung Cancer**																
No	727	132	1	—		1	—		805	54	1	—		1	—	
Yes	25	16	3.52	1.83	6.78	2.69	1.27	5.72	26	15	8.60	4.30	17.19	8.83	3.81	20.44
**Metabolic Syndrome** ^e^																
No	707	140	1	—		1	—		784	63	1	—		1	—	
Yes	45	8	0.90	0.41	1.95	0.90	0.39	2.07	47	6	1.59	0.65	3.86	2.45	0.94	6.40

^a^ “None” denotes 0 COPD admissions the following year. “≥1” denotes at least one COPD admission the following year.

^b^ Odds ratios and 95% confidence intervals: “ORc” denotes crude odds ratio

^c^ “ORa” denotes adjusted OR by age, sex, smoking status, COPD severity (GOLD Grades 1–4) and frequent exacerbator phenotype (yes/no) the previous year.

^d^ Any severe heart disease: ischemic heart disease, heart failure, atrial fibrillation.

^e^ Metabolic syndrome: BMI ≥30, diabetes mellitus, high blood pressure, use of statins as “subrogate” of dyslipidemia.

^f^ Overall mortality for all causes

S2 Table presents an analysis of the existence of future severe exacerbations with the regression results of all the main associated variables in the full model. The associations and statistically significance were maintained.

## Discussion

Regarding sociodemographic and lifestyle factors, older age was associated with new hospitalized exacerbations and mortality the following year. As performed in the largest prospective cohort study (ECLIPSE), we ordinally categorized age into 10-year increases. Our results for the risk of hospitalized exacerbations (OR, 1.35) are similar to those of ECLIPSE (OR, 1.29) [14,18]. With respect to mortality, most published studies also show similar results [22,23,24,25,26].

A prior history of hospitalized exacerbations of COPD was associated with new hospitalized exacerbations and showed a statistically significant dose-response pattern. This effect was independent of COPD severity and the main confounders identified in the present study. This finding is in line with those of other international studies [16,27,28,29] and the ECLIPSE study. In the latter study, at least one hospitalized exacerbation the previous year was the main predictor of at least one hospitalized exacerbation over the following 2 years with a hazard ratio of 2.71 (95%CI, 2.24–3.29) [18].

Independent of the severity of airflow limitation, a history of hospitalized exacerbations the previous year was associated with reduced survival in the present study. This is also supported by the findings of the ECLIPSE [18] and other international studies [23,30].

Regarding COPD severity, an important difference between the present study and that of ECLIPSE is that we included GOLD grade 1 patients, whereas ECLIPSE did not (FEV1<80%). In our sample, 10.4% of the included patients were GOLD grade 1; 9.3% of these mild GOLD grade 1 patients developed at least one exacerbation requiring hospital admission. These results present an original clinical perspective, supporting the hypothesis that even with milder forms of COPD, there may be some susceptibility to severe exacerbations. In the present study, as in the ECLIPSE cohort study, the incidence of exacerbations requiring hospital admission increased according to disease severity; it was 44.0% in the very severe GOLD grade 4 patients in our study. Thus, COPD severity was also associated with exacerbations requiring hospitalization in our crude and adjusted models, which supports the findings of previous studies [15,18,25,31,32]. When we added the frequent exacerbator phenotype to the multivariable model, the adjusted *p* trend and associations remained statistically significant. This suggests that the effect of COPD severity is independent of the frequent exacerbator phenotype.

A GOLD grade 3 or 4 (severe or very severe underlying COPD) is an indication for hospital admission according to GOLD guideline [11]. That is also the case with the Spanish GesEPOC guideline [33]. This could explain the associations related to hospitalization.

Regarding mortality, the independent predictive accuracy for COPD severity according to our results was lower than the association between COPD severity and hospitalization; it only appeared to be clinically important for the most advanced severity grade (very severe GOLD grade 4). This lower predictive accuracy has been described in other studies [22,24,34,35,36].

In the present study, several comorbidities, such as heart failure and diabetes, were found to be associated with both hospitalized exacerbations and mortality. On the one hand, it is plausible that with greater number and severity of the comorbidities, more interactions will increasingly result in poor health. Worsening of the general health status or comorbidities could, for example, occur as a consequence of COPD exacerbation treatment side effects (systemic steroids inducing hyperglycemia or muscle weakness) [37,38]. On the other hand, in both the GOLD [11] and Spanish GesEPOC guidelines [33], the existence of severe comorbidities is also an indication for admission. Anyway, the importance of comorbidities in the role of hospitalized exacerbations and mortality has grown in recent years [11,26,33,39,40].

As a main limitation of the present study, it is necessary to note the retrospective design based on secondary information through clinical databases that were not specifically designed for research objectives. In retrospective studies based on secondary information (records), a main limitation could be the low quality of that information; this could be due either to insufficient completion of medical records or lack of agreement among different records. To minimize bias in the study protocol, we chose only the variables that had been more homogeneously, systematically, and objectively collected in the records. Where possible, we obtained agreement by making a comparison between the primary-care and hospital records.

With some important variables, such as the FEV1 quantitative results (disease severity as defined by a spirometric assessment of lung function according to the GOLD guideline), we found a non-negligible proportion of missing data (N = 177, 19.7%). We specifically studied the performance of these missing values; we did so by treating missing values as a separate category and comparing the association between the missing values and risk of hospitalized exacerbations and overall mortality. The missing values showed greater similarity to the reference category (mild GOLD grade 1) than to the more severe GOLD grades 2–4. This suggests that if those missing values were not actually unknown but were mild GOLD grade 1, similar OR for the remaining GOLD grades 2–4 would have been obtained with a more precise 95%CI. If the missing data had actually not been mild GOLD grade 1, the OR would have changed with more precise 95%CI.

Another caveat concerning GOLD severity measurement relates to the age of the last data registered. Because this is an observational study, we were able to obtain FEV1 only from the last spirometry registered for each patient. According to the registered data, FEV1 values were available for 70% of the patients for the previous 3 years (504 of 723 patients). When we conducted a sensitivity analysis on those 504 patients, the findings were consistent with our previous results. Evidence from recently published studies indicates that the decline in FEV1 values was considerably less than anticipated [41,42,43,44].

Individual follow-up studies using mortality as the event would demand a large sample size to obtain sufficient statistical power. The present study lacked statistical power to identify significant associations for mortality among the different COPD severity grades. A future meta-analysis including more individual studies could determine such associations with greater precision.

External validity is one of the main classical limitations of clinical trials [45,46]. This problem can also affect observational studies based on strict inclusion and exclusion criteria [47]. We confirmed that our final analyzed sample was representative regarding age, sex, and primary health-care centers with respect to the base population. A major strength of the present study is that our sample was a population-based (real-life) sample. Accordingly, mild grades and nonsmokers with confirmed COPD were identified and included in the simple random sampling. The prevalence of 15.7% of confirmed COPD among never smokers is supported by other studies [2,3,4].

Our data were based on records. Thus, it is improbable that bias arose through lack of blindness among patients’ care providers (they treated the patients blinded retrospectively before the development of the study). To minimize selection bias, we chose only confirmed COPD cases. We attempted to obtain the independent effect of predictors in our epidemiological and statistical approach by controlling confounding.

## Conclusions

Despite its limitations, the present study supports the following hypothesis: independent of COPD severity, a history of hospitalized exacerbations the previous year is associated with new hospitalized exacerbations and mortality the following year. Of the mild GOLD grade 1 patients, 9% developed at least one exacerbation requiring hospital admission the following year. This finding may have important implications for the management and monitoring of milder forms of COPD patients. COPD severity was also independently associated with new hospitalized exacerbations the following year. Older age and several comorbidities, such as heart failure and diabetes, were associated with both hospitalized exacerbations and mortality.

## Supporting Information

S1 Table Baseline clinical characteristics (co-morbidities) of the patients. ^a^ None” denotes 0 COPD admissions the following year. “≥1” denotes at least one COPD admission the following year. ^b^ Any severe heart disease: ischemic heart disease, heart failure, atrial fibrillation.^c^ Metabolic syndrome: BMI ≥30, diabetes mellitus, high blood pressure, use of statins as “subrogate” of dyslipidemia.
(DOCX)

S2 Table Analysis of the existence of future severe exacerbations, with the regression results of all the main associated variables in the full model. ^a^ Odds Ratios adjusted for all the variables above, as well as sex and smoking status.
(DOCX)
