# Supplementary material for: Predictors of Hospitalized Exacerbations and Mortality in Chronic Obstructive Pulmonary Disease
Source: PLoS One. 2016 Jun 30;11(6):e0158727. doi: 10.1371/journal.pone.0158727 (PMC4928940; doi:10.1371/journal.pone.0158727)
Supplement: S1 Table — a None” denotes 0 COPD admissions the following year. “≥1” denotes at least one COPD admission the following year. b Any severe heart disease: ischemic heart disease, heart failure, atrial fibrillation.c Metabolic syndrome: BMI ≥30, diabetes mellitus, high blood pressure, use of statins as “subrogate” of dyslipidemia. (DOCX) [file pone.0158727.s001.docx]

S1 Table. Baseline clinical characteristics (co-morbidities) of the patients.

|  | **Hospìtalized Exacerbations** | | | | | | |
| --- | --- | --- | --- | --- | --- | --- | --- |
|  | **None^a^** |  | **≥1** |  | **Total** |  |  |
|  | **N=752** | **row %** | **N=148** | **row %** | **N=900** | **Column%** | ***p value*** |
| **Co-morbidities** |  |  |  |  |  |  |  |
| None | 126 | 93.3% | 9 | 6.7% | 135 | 15% | *0.001* |
| At least one of the co-morbidities below | 626 | 81.8% | 139 | 18.2% | 765 | 85% |  |
| **Atrial Fibrillation** |  |  |  |  |  |  |  |
| No | 626 | 86.3% | 99 | 13.7% | 725 | 80.6% | *<0.001* |
| Yes | 126 | 72.0% | 49 | 28.0% | 175 | 19.4% |  |
| **Ischemic heart disease** |  |  |  |  |  |  |  |
| No | 632 | 85.1% | 111 | 14.9% | 743 | 82.6% | *0.008* |
| Yes | 120 | 76.4% | 37 | 23.6% | 157 | 17.4% |  |
| **Heart Failure** |  |  |  |  |  |  |  |
| No | 632 | 87.5% | 90 | 12.5% | 722 | 80.2% | *<0.001* |
| Yes | 120 | 67.4% | 58 | 32.6% | 178 | 19.8% |  |
| **Any severe heart disease ^b^** |  |  |  |  |  |  |  |
| No | 498 | 89.6% | 58 | 10.4% | 556 | 61.8% | *<0.001* |
| Yes | 254 | 73.8% | 90 | 26.2% | 344 | 38.2% |  |
| **High Blood Pressure** |  |  |  |  |  |  |  |
| No | 303 | 84.6% | 55 | 15.4% | 358 | 39.8% | *0.477* |
| Yes | 449 | 82.8% | 93 | 17.2% | 542 | 60.2% |  |
| **Diabetes** |  |  |  |  |  |  |  |
| No | 559 | 85.7% | 93 | 14.3% | 652 | 72.4% | *0.004* |
| Yes | 193 | 77.8% | 55 | 22.2% | 248 | 27.6% |  |
| **Osteoporosis** |  |  |  |  |  |  |  |
| No | 685 | 83.9% | 131 | 16.1% | 816 | 90.7% | *0.325* |
| Yes | 67 | 79.8% | 17 | 20.2% | 84 | 9.3% |  |
| **Psiquiatric History** |  |  |  |  |  |  |  |
| No | 503 | 84.8% | 90 | 15.2% | 593 | 65.9% | *0.154* |
| Yes | 249 | 81.1% | 58 | 18.9% | 307 | 34.1% |  |
| **Lung Neoplasm** |  |  |  |  |  |  |  |
| No | 727 | 84.6% | 132 | 15.4% | 859 | 95.4% | *<0.001* |
| Yes | 25 | 61.0% | 16 | 39.0% | 41 | 4.6% |  |
| **Metabolic Syndrome^c^** |  |  |  |  |  |  |  |
| No | 707 | 83.5% | 140 | 16.5% | 847 | 94.1% | *0.785* |
| Yes | 45 | 84.9% | 8 | 15.1% | 53 | 5.9% |  |
