# Supplementary material for: Predictors of Hospitalized Exacerbations and Mortality in Chronic Obstructive Pulmonary Disease
Source: PLoS One. 2016 Jun 30;11(6):e0158727. doi: 10.1371/journal.pone.0158727 (PMC4928940; doi:10.1371/journal.pone.0158727)
Supplement: S2 Table — a Odds Ratios adjusted for all the variables above, as well as sex and smoking status. (DOCX) [file pone.0158727.s002.docx]

S2 Table. Analysis of the existence of future severe exacerbations, with the regression results of all the main associated variables in the full model.

|  | **Hospìtalized Exacerbations** | |  |  |  |
| --- | --- | --- | --- | --- | --- |
|  | **None ^a^** | **≥1** |  |  |  |
|  | **N=752** | **N=148** | **ORa^a^** | **(95%** | **CI)** |
| **Age** continuous (per 1-year increase) | -- | -- | 1.02 | 1.00 | 1.05 |
| **Number of severe exacerbations (requiring Hospital admission) the previous year** |  |  |  |  |  |
| None (0 COPD Admissions) | 649 | 81 | 1 | -- |  |
| 1 COPD Admission | 79 | 36 | 2.87 | 1.71 | 4.83 |
| ≥2 COPD Admissions | 24 | 31 | 5.18 | 2.67 | 10.06 |
| *Linear p trend* |  |  | *<0.001* |  |  |
| **COPD severity according to FEV1** | | |  |  |  |
| FEV1 - Mild-GOLD Grade 1 (reference category) | 68 | 7 | 1 | -- |  |
| FEV1 - Moderate-GOLD Grade 2 | 363 | 70 | 1.23 | 0.51 | 2.96 |
| FEV1 - Severe-GOLD Grade 3 | 146 | 44 | 1.56 | 0.62 | 3.92 |
| FEV1- Very Severe-GOLD Grade 4 | 14 | 11 | 4.14 | 1.22 | 14.04 |
| *Linear p trend* |  |  | *0.018* |  |  |
| **Heart Failure** |  |  |  |  |  |
| No | 632 | 90 | 1 | -- |  |
| Yes | 120 | 58 | 2.36 | 1.48 | 3.75 |
| **Diabetes** |  |  |  |  |  |
| No | 559 | 93 | 1 |  |  |
| Yes | 193 | 55 | 1.64 | 1.05 | 2.56 |
